# Photovoltaic and Impedance Spectroscopy Study of Screen-Printed TiO_2_ Based CdS Quantum Dot Sensitized Solar Cells

**DOI:** 10.3390/ma8010355

**Published:** 2015-01-19

**Authors:** M. Atif, W. A. Farooq, Amanullah Fatehmulla, M. Aslam, Syed Mansoor Ali

**Affiliations:** 1 Department of Physics and Astronomy, College of Science, King Saud University, P.O. Box 2455, Riyadh 11451, Saudi Arabia; E-Mails: wafarooq@hotmail.com (W.A.F.); aman@ksu.edu.sa (A.F.); muhd.aslam@gmail.com (M.A.); mansoor_phys@yahoo.com (S.M.A.); 2 National Institute of Laser and Optronics, Nilore, 45650 Islamabad, Pakistan

**Keywords:** cadmium sulphide (CdS), quantum dot, solar cells, photovoltaic, impedance spectroscopy

## Abstract

Cadmium sulphide (CdS) quantum dot sensitized solar cells (QDSSCs) based on screen-printed TiO_2_ were assembled using a screen-printing technique. The CdS quantum dots (QDs) were grown by using the Successive Ionic Layer Adsorption and Reaction (SILAR) method. The optical properties were studied by UV-Vis absorbance spectroscopy. Photovoltaic characteristics and impedance spectroscopic measurements of CdS QDSSCs were carried out under air mass 1.5 illuminations. The experimental results of capacitance against voltage indicate a trend from positive to negative capacitance because of the injection of electrons from the Fluorine doped tin oxide (FTO) electrode into TiO_2_.

## 1. Introduction

For the development of next generation solar cells quantum dot-sensitized solar cells (QDSSCs) have attracted great attention due to their simplicity and low cost [1,2]. The developments involve progress in nanotechnology especially the application and synthesis of nanomaterials facilitates for the development of QDSSCs. Researchers around the world are trying to improve the performance of QDSSCs focusing on fundamental issues such as optimization of the device structure by advanced processing methods and improving the understanding of device physics [3,4,5,6,7,8,9,10,11,12,13]. Metal chalcogenide quantum dots (QDs) (such as CdSe, CdS, Ag_2_S, PbS, CdTe and Bi_2_S_3_) usually serve as sensitizers in QDSSCs due to their absorption in the visible region [14,15,16,17,18,19,20,21]. The advantages of QD sensitizers over conventional dyes are their large intrinsic dipole moment, multiple exciton generation by impact ionization and higher extinction coefficients [22,23,24]. The commonly used QD sensitizer is cadmium sulfide (CdS) due to its large optical absorption in the visible range and proper band alignment between CdS and TiO_2_.

The best choice in semiconductor oxides is titanium dioxide (TiO_2_) in QDSSCs which is due to its availability in the market at compatible price as well as its good biocompatibility, and nontoxicity. The most commonly used deposition processes to prepare TiO_2_ films are doctor blading and screen-printing techniques. A widespread industrially-applied method is screen-printing of TiO_2_ due to its uniform morphologies, controllable thickness, and fast production.

Due to excellent catalytic activity and good electric conductivity Pt is normally used as counter electrode in QDSSCs. It is important to mention that Pt is a rare metal which is very expensive. Researchers around the world are trying to develop counter electrodes by using different inexpensive carbonaceous materials such as graphite, carbon black, activated carbon, hard carbon sphere, carbon nanotube, fullerene and graphene, having high electrochemical activity [25,26,27].

In the present research we report the characterization of CdS quantum dot sensitized solar cells based on screen-printed TiO_2_. A screen-printing technique is used to prepare the TiO_2_ film. The CdS QDs solar cell was synthesized using screen-printed TiO_2_ thin film and the successive ionic layer adsorption and reaction deposition (SILAR) method.

## 2. Experimental Details

### 2.1. Sample Preparation

In a typical synthesis, the substrate was ultrasonically cleaned sequentially in acetone, isopropyl alcohol (IPA), and deionized (DI) water for 15 min each solvent and finally dried under N_2_ flow. Titanium (IV) oxide (a mixture of rutile and anatase) nanoparticles paste (700355) was purchased from Sigma Aldrich (St. Louis, MO, USA). This paste was further diluted with ethanol and used for the film preparation on glass substrates by a screen printing technique. Initially an absorption layer of about 3 μm was deposited onto the Fluorine doped tin oxide (FTO) substrates by a homemade screen-printer using this paste. The printed glass slides were then placed on a hot plate at 120 °C for 15 min and then allowed to cool down to room temperature (RT). This process was repeated three times to get a ~9 nm thick film on the substrate. The multilayer films obtained on the glass substrate were finally annealed at 450 °C for 1 h. The printed glass slides were then cut into small 2 × 2.5 cm^2^ working electrodes which contained 0.1256 cm^2^ of TiO_2_ prints.

Cadmium sulphide (CdS) quantum dots were placed on the three layered mesoporous TiO_2_ film by the Successive Ionic Layer Adsorption and Reaction (SILAR) method. Then this film was dipped for 5 min into a 0.5 M cadmium nitrate [Cd (NO_3_)_2_] ethanol solution (cadmium cationic precursor) and rinsed with ethanol, heated for 10 min, cooled to room temperature and then dipped for another 5 min into a 0.5 M sodium sulfide [Na_2_S] water solution (sulfur anionic precursor) and rinsed again with water, heated for 10 min, and cooled to room temperature. This cadmium sulphide adsorbed TiO_2_ film was dried with a N_2_ air stream. The two-step dipping procedure is known as a 1 SILAR cycle and this process is continued for 10 cycles. We did not measure the amount of CdS on TiO_2_ but to increase the amount of CdS QDs, the number of the SILAR cycles is increased which leads to a substantial red shift indicating a decrease of the band gap. Moreover limited SILAR cycles possess a quantum confinement effect and with the increase of SILAR cycles there is an enhancement in the light absorption. This result is in good agreement with the previous reported studies [28]. Counter electrodes were developed by sputtering platinum on a FTO substrate.

Polysulfide electrolytes were prepared by mixing suitable quantities of 0.5 M Na_2_S, 2 M S, and 0.2 M KCl powders in water/methanol solution taken in the ratio 3/7.

### 2.2. Characterization

A Keithley 4200 semiconductor characterization system (Keithley Instruments, Solon, OH, USA) was used for the photovoltaic characterization and impedance measurements. Photovoltaic measurements were performed using a small area solar simulator model SASS (PV measurements, Boulder, CO, USA) while intensity was measured using a TM-206 solar power meter (Tenmars, Taipei, Taiwan). All the experimental results were recorded at room temperature.

## 3. Results and Discussion

### 3.1. Absorption Studies

**Figure 1:**
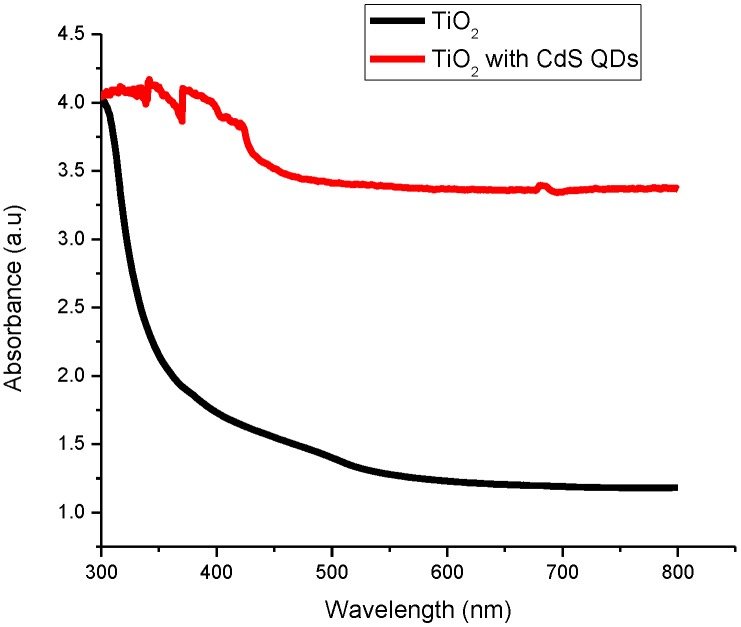
UV-Vis absorption spectra of titanium dioxide (TiO_2_) and cadmium sulfide quantum dot (CdS QD) based on screen-printed TiO_2_.

Figure 1 shows the absorption spectra of TiO_2_ and CdS QD deposited on screen-printed TiO_2_. In the UV-Vis spectra of CdS QD based on screen-printed TiO_2_, the absorption shoulder peak around 350 nm is allocated to the optical transition of the exciton in the first excitonic state and the substantial blue shift in this peak with respect to bulk CdS (431 nm) relates to the formation of CdS [29]. It is evident from the experimental results that CdS QD based on screen-printed TiO_2_ indicates a wide absorption spectrum in the UV-Vis region and shows significant absorption.

### 3.2. TEM Images

**Figure 2:**
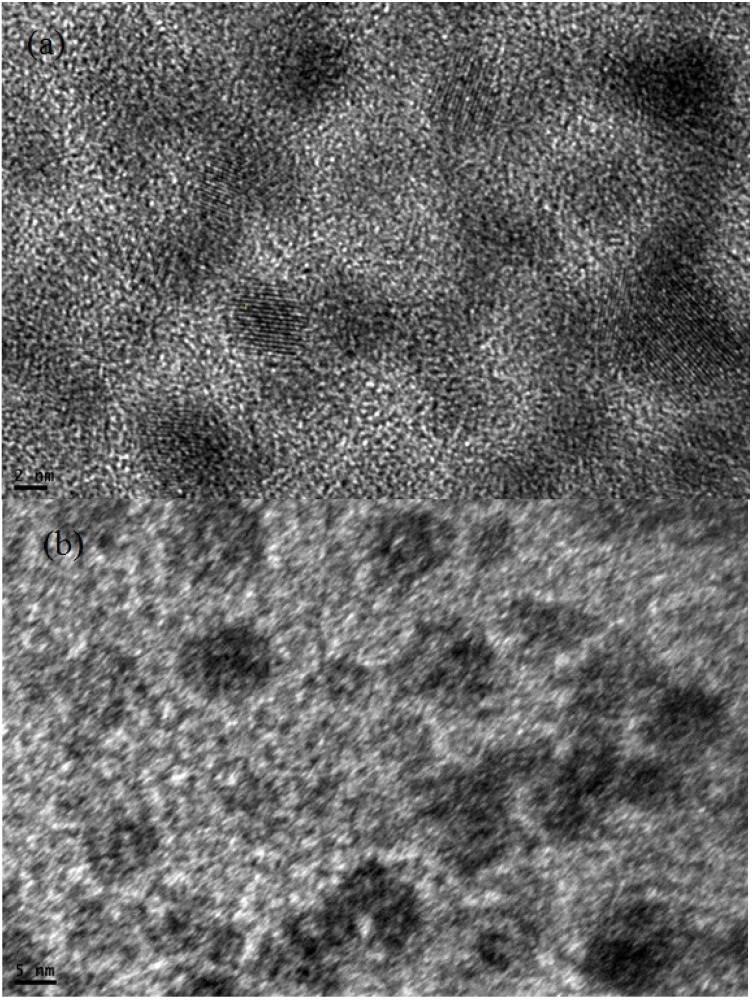
TEM images of CdS QD (**a**) low resolution (**b**) high resolution.

TEM images of CdS QDs deposited by the SILAR method on the TiO_2_ nanostructure at low and high magnification are shown in Figure 2a,b. From TEM morphology, the size of CdS QDs on the surface of TiO_2_ nanostructure is observed to be 2–5 nm.

### 3.3. Photovoltaic Characteristics

The equation for a solar cell representing the open circuit voltage [30,31,32] is:

(1) $$V_{oc}=\frac{nkT}{q}\text{ln}\left(\frac{J_{sc}}{J_{o}}+1\right)$$

where *n* is the diode ideality factor, *k* the Boltzmann’s constant, *q* the electric charge and *J_0_* is the reverse saturation current density.

**Figure 3:**
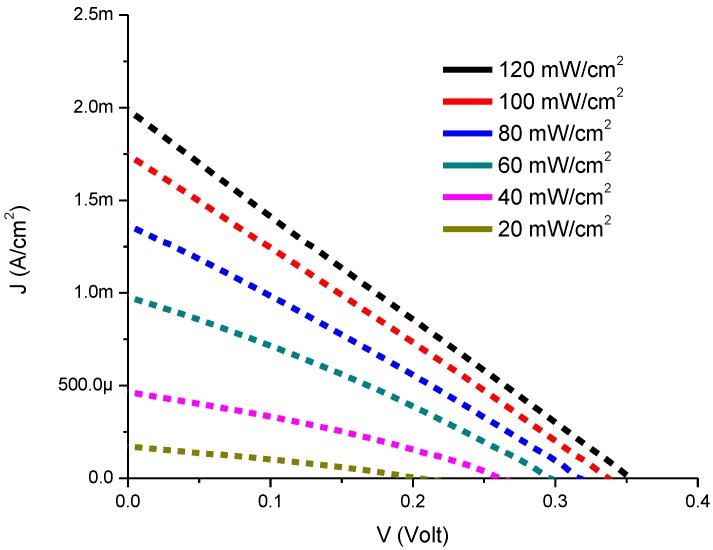
Current-Voltage characteristics of CdS QD based on screen-printed TiO_2_.

Figure 3 represents the experimental results for current-voltage characteristics of CdS QD deposited on screen-printed TiO_2_. It is clear from the experimental data that the photocurrent and photovoltage values increase with increasing illumination intensities. We found improved current density values with CdS QD based on screen-printed TiO_2_ which confirms earlier reports of increase in electrical conduction. The plot of current *vs.* voltage exhibits a photo lateral collecting effect which is responsible for almost parallel shift with linearity [33,34].

**Figure 4:**
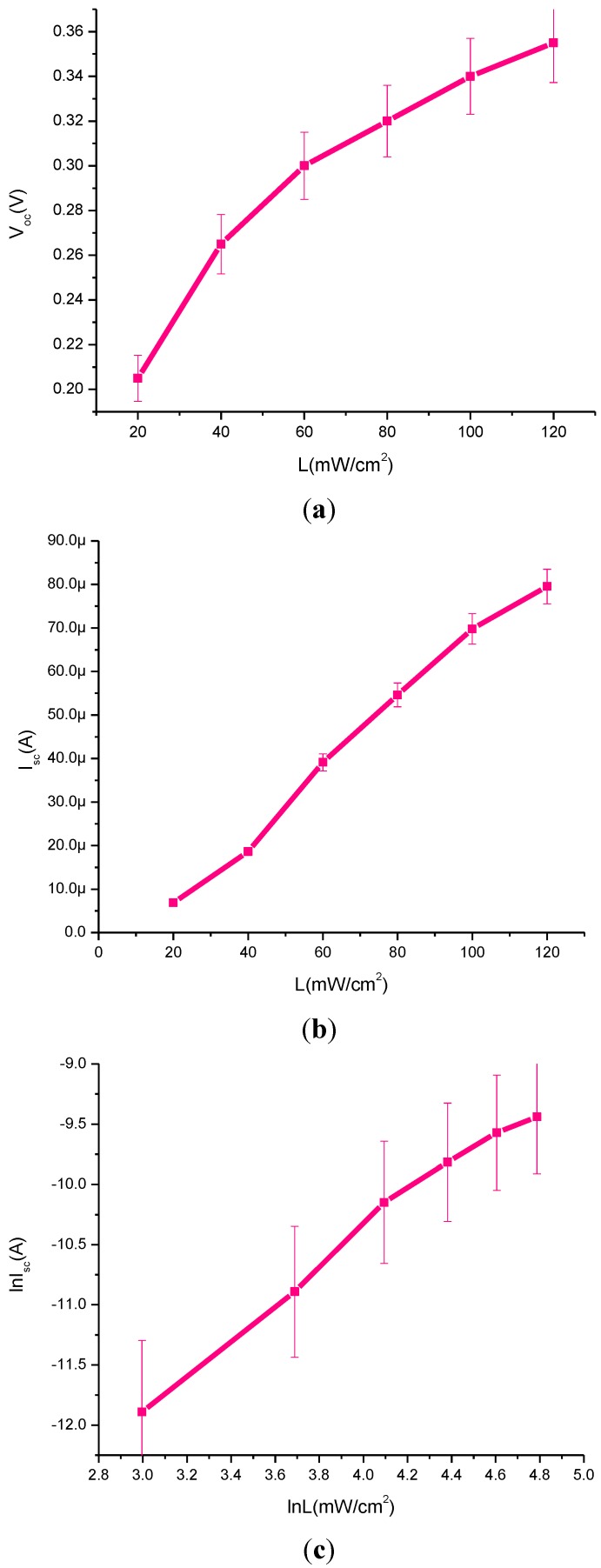
Plots of (**a**) *V*_oc_ *vs.* light intensity, (**b**) *I*_sc_ *vs.* light intensity and (**c**) ln(*I_SC_*) *vs.* ln(*L*).

Figure 4 demonstrates the following plots; *V_oc_* against light intensity, Figure 4a; *I_sc_* against light intensity, Figure 4b; and ln (*I_SC_*) against ln (*L*), Figure 4c. The experimental data recorded for CdS QD based on screen-printed TiO_2_ clearly shows a substantial increase in the *V_oc_* observed with the increase of light intensity. It also shows an increasing trend in *I_sc_* and ln (*I_SC_*) with the increase of light intensity.

The fill factor is calculated using the formula [35,36]:

(2) $$\text{FF}=\frac{V_{\text{mpp}}J_{\text{mpp}}}{V_{oc}J_{sc}}=\frac{P_{\text{max}}}{V_{oc}J_{sc}}$$

Normally there are two dominant losses for the Fill Factor (FF). One is due to high series resistance and the other due to low shunt resistance. In the present study the cause of low FF is a low shunt resistance which means that increasing the light intensity for a fixed voltage will lead to a smaller fraction of the light-generated current flowing through the shunt [37]. The observed increasing trend is likely due to the presence of CdS QD in the TiO_2_ matrix which helps in the light harvesting.

**Figure 5:**
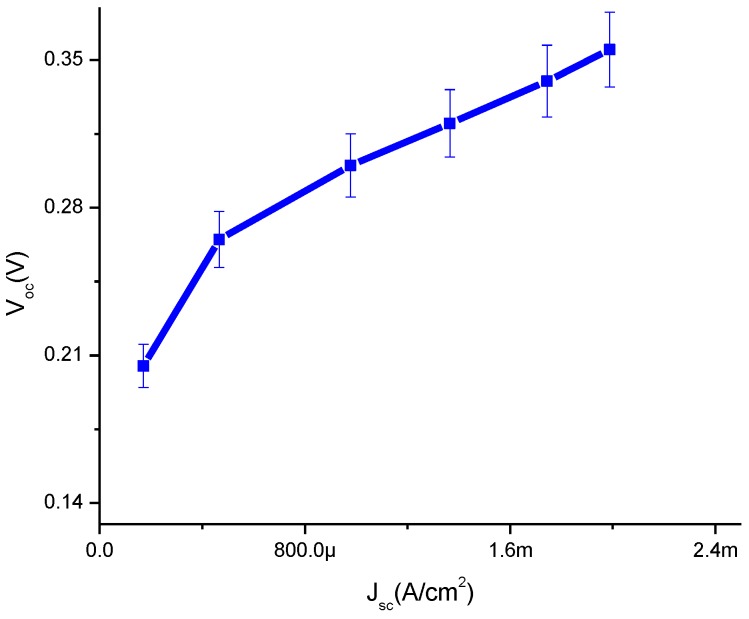
Plot of open circuit voltage against current density.

In Figure 5, it is evident that in the presence of CdS QD based on screen-printed TiO_2_ the *V_OC_* shows an increasing trend with the increase of *J_SC_* which means that it indicates linear behavior. The shunt resistance is low, typically due to fabrication defects and it provides power losses in solar cells by providing an alternate current path for the light-generated current. The main reason is that it reduces the amount of current flowing through the solar cell junction and also the voltage from the solar cell. It is observed that especially at low levels, the light-generated current is low. These current losses to the shunt resulted in a greater impact and it was also observed at lower voltages that the effective resistance of the solar cell is high.

Where the maximum power point is the product of current density and voltage (*J*_mpp_ × *V*_mpp_) at each illumination intensity:

(3) $$P_{\text{max}}=J_{\text{mpp}}\times V_{\text{mpp}}$$

**Figure 6:**
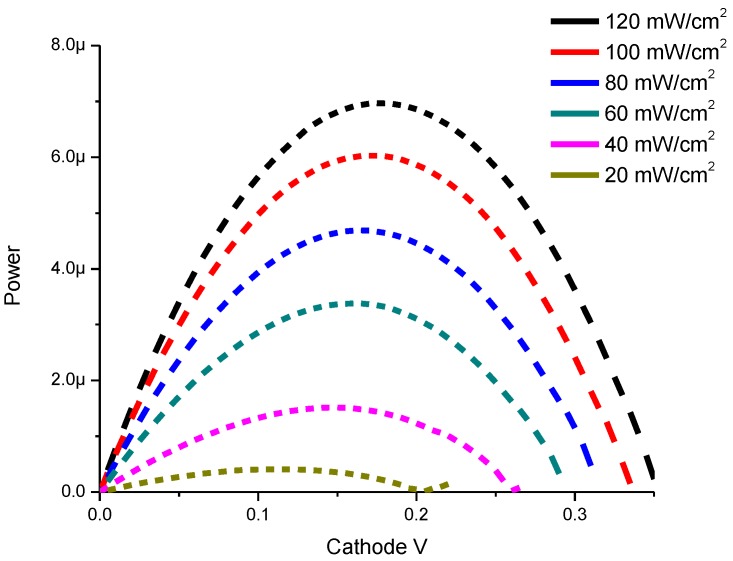
Power-Voltage characteristics of CdS QD based on screen-printed TiO_2_.

Power-Voltage characteristics of CdS QD based on screen-printed TiO_2_ are shown in Figure 6.

The experimental results in Figure 6 show that the maximum power peak is moved to higher voltages if we increase the incident light as follows: 0.26 V, 1.5 μW at 20 mW/cm^2^ and 0.33 V, 6.0 μW at 100 mW/cm^2^.

### 3.4. Impedance Characteristics

#### 3.4.1. Capacitance-Voltage Characteristics

**Figure 7:**
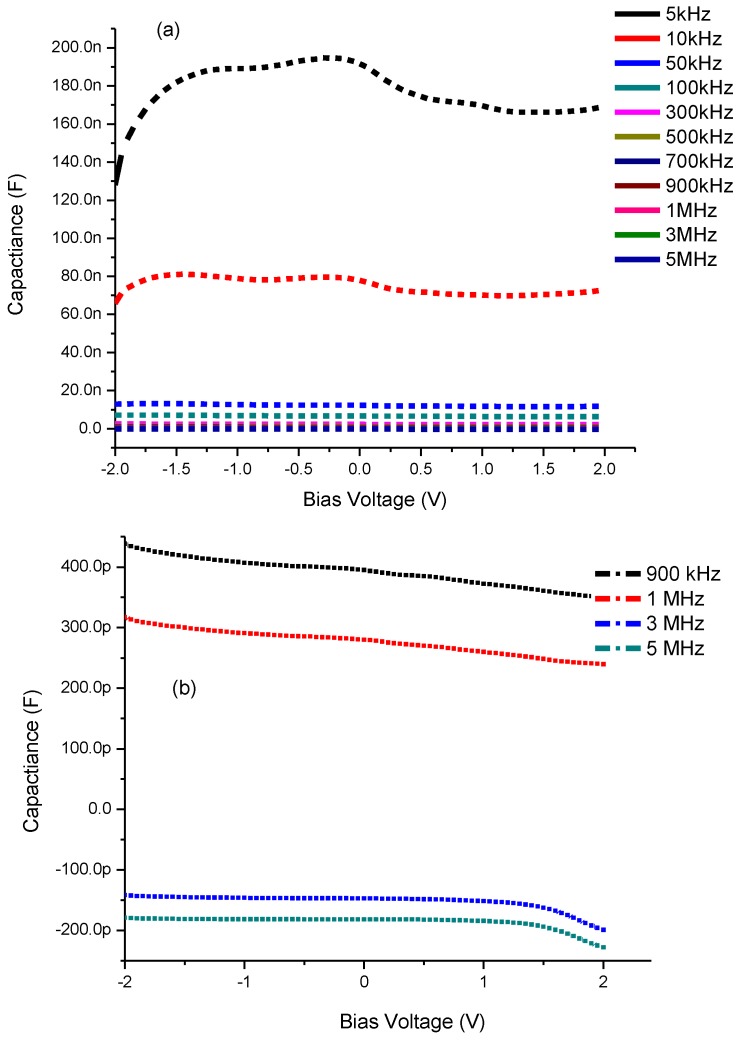
Capacitance-Voltage characteristics of CdS QD based on screen-printed TiO_2_ (**a**) at all frequencies and (**b**) negative capacitance after 1 MHz frequency.

It is observed in Figure 7a,b of CdS QD based on screen-printed TiO_2_ that by increasing the bias voltage from −2.0 to +2.0 V, the capacitance also shows an increasing trend and this continues until it reaches its maximum value. Thereafter it shows a decrease in its value leading towards saturation followed by an increase in bias voltages 1.20 and 1.46 V for 5 and 10 kHz frequencies respectively. However when we increase the frequency from 50 kHz to 5 MHz the capacitance shows a decreasing behavior towards zero and even going to negative capacitance after 1 MHz frequency (Figure 7b). This trend from positive to negative capacitance, also called inductive behavior, is very common in several materials which means the current lags behind the voltage [36]. Such a change is considered to be due to the injection of electrons from the FTO electrode into TiO_2_ [38].

#### 3.4.2. Conductance-Voltage Characteristics

**Figure 8:**
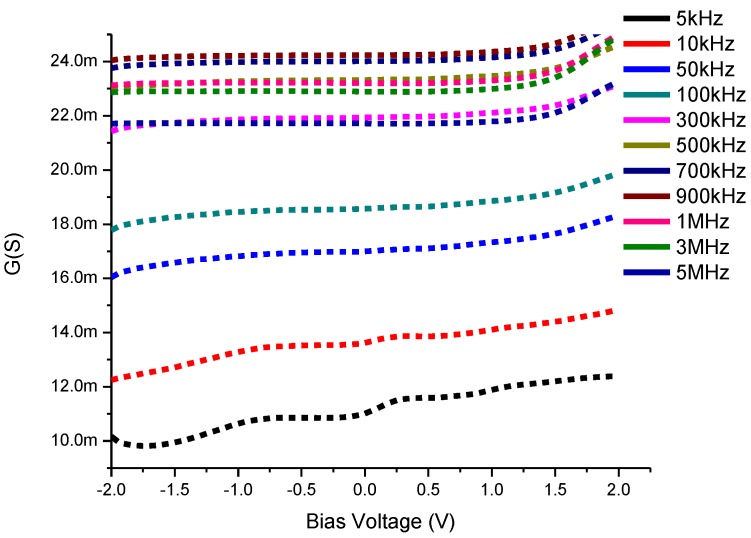
Conductance-Voltage (G-V) characteristics of CdS QD based on screen-printed TiO_2_.

This technique of analysis is based on the losses of conductance between the interface states and the majority carrier band of the semiconductor due to the exchange of majority carriers at a small ac signal applied equal to 20 mV in the current study [39] of the semiconductor devices. Figure 8 shows the conductance-voltage (G-V) characteristics of CdS QD based on screen-printed TiO_2_. The conductance represents an increase with the increase of the applied frequency up to 900 kHz and is followed by a decreasing trend in its values which means this trend is slowed down with the change of capacitance from positive values to negative values at higher values of the frequency such as 1, 3, 5 MHz.

#### 3.4.3. Resistance-Voltage Characteristics

*R_s_* (series resistance) is an important parameter to predict noise ratio of a device in terms of frequency [40]. Hence it is very important to indicate at different voltages the values of *R_s_* for the total range of frequency investigated. The formula for voltage and frequency dependent series resistance of the solar cell is as follows which is taken from the experimental data of *C-V-f* measurements [41]:

(4) $$R_{s}=(\frac{G_{MA}}{G_{MA}^{2}+w^{2}C_{MA}^{2}})$$

where *C*_MA_ and *G*_MA_ are the measured values of capacitance and conductance respectively.

**Figure 9:**
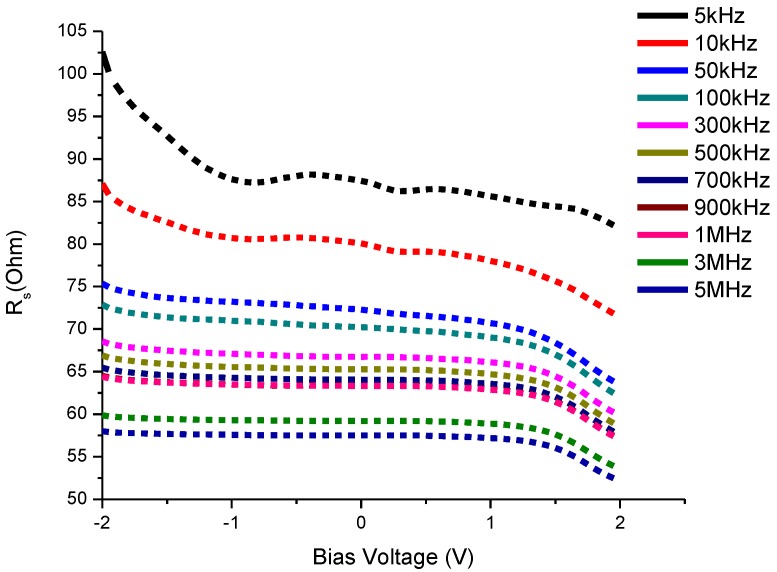
Resistance-Voltage (Rs-V) characteristics of CdS QD based on screen-printed TiO_2_.

It is evident from Figure 9 that the decrease in series resistance is gradual from lower to higher frequencies at bias voltages from −2 to +2 V. This behavior is almost similar for all CdS QDs based on screen-printed TiO_2_.

## 4. Conclusions

We fabricated cadmium sulphide (CdS) quantum dots onto the screen-printed TiO_2_ photoanode using the SILAR method. The TEM image indicated that the average diameter of the CdS quantum dot was 2–5 nm. The fabricated solar cell in the configuration of FTO/TiO_2_/CdS QD/Pt/FTO measured the short circuit current and open circuit voltage values 1.75 mA/cm^2^ and 0.33 V respectively at AM 1.5 light intensity. The maximum power value moved to higher voltages with the increase of incident light: 0.26 V, 1.5 μW at 20 mW/cm^2^ and 0.33 V, 6.0 μW at 100 mW/cm^2^. The investigation of Impedance spectroscopy of the cell with C-V, G-V and R_s_-V plots showed the dependency of frequency varied between 5 kHz to 5 MHz in the bias voltage range from −2 to +2 V. The C-V measurements of a screen-printed TiO_2_ CdS quantum dot sensitized solar cell demonstrate an inductive behavior of capacitance above 1 MHz frequency as evident from the negative capacitance values. The negative capacitance values also indicate a very high value of conduction at high frequencies. This behavior is due to the injection of electrons from the FTO electrode into TiO_2_.
